# Machine learning-guided differential gene expression analysis identifies a highly-connected seven-gene cluster in triple-negative breast cancer

**DOI:** 10.37796/2211-8039.1467

**Published:** 2024-12-01

**Authors:** Hany Ghazal, El-Sayed A. El-Absawy, Waleed Ead, Mohamed E. Hasan

**Affiliations:** a Bioinformatics Department, Genetic Engineering and Biotechnology Research Institute, University of Sadat City, Sadat City, Egypt; b Information Systems Department, Faculty of Computers and Artificial Intelligence, Beni-Suef University, Beni-Suef, Egypt

**Keywords:** Precision medicine, Triple-negative breast cancer, Artificial intelligence, Biomarkers, Stratification

## Abstract

**Background:**

One of the most challenging cancers is triple-negative breast cancer, which is subdivided into many molecular subtypes. Due to the high degree of heterogeneity, the role of precision medicine remains challenging. With the use of machine learning (ML)-guided gene selection, the differential gene expression analysis can be optimized, and eventually, the process of precision medicine can see great advancement through biomarker discovery.

**Purpose:**

Enhancing precision medicine in the oncology field by identification of the most representative differentially-expressed genes to be used as biomarkers or as novel drug targets.

**Methods:**

By utilizing data from the Gene Expression Omnibus (GEO) repository and The Cancer Genome Atlas (TCGA), we identified the differentially expressed genes using the linear model for microarray analysis (LIMMA) and edgeR algorithms, and applied ML-based feature selection using several algorithms.

**Results:**

A total of 27 genes were selected by merging features identified with both LIMMA and ML-based feature selection methods. The models with the highest area under the curve (AUC) are CatBoost, Extreme Gradient Boosting (XGBoost), Random Forest, and Multi-Layer Perceptron classifiers. ESR1, FOXA1, GATA3, XBP1, GREB1, AR, and AGR2 were identified as hub genes in a highly interconnected cluster.

**Conclusion:**

ML-based gene selection shows a great impact on the identification of hub genes. The ML models built can improve precision oncology in diagnosis and prognosis. The identified hub genes can serve as biomarkers and warrant further research for potential drug target development.

## 1. Background

Triple Negative Breast Cancer (TNBC) is the type of breast cancer that is negative for all three hormonal receptors, ER, PR, and HER2 [1,2] and is characterized by a poor prognosis. TNBC accounts for approximately 15–20% of all breast cancers and is more common in young women below 40 and women of African or Hispanic descent [3].

Based on gene expression studies, TNBC is classified into six subtypes: basal-like 1 (BL1) and basal-like 2 (BL2), mesenchymal (M), mesenchymal stem-like (MSL), immunomodulatory (IM), and luminal androgen receptor (LAR) subtypes [1,4,5]. Recent studies have refined these six molecular subtypes into four: BL1, BL2, M, and LAR, which have significant implications for diagnosis, treatment, and precision medicine [6].

Identifying differentially expressed genes and conducting functional and pathway enrichment analysis are cornerstones in discovering new drug targets, designing new drugs, or repurposing existing ones [4,7,8]. Understanding the molecular and genomic signature of each TNBC subtype helps precision oncology deliver its message in this context [9].

BL1 subtypes are associated with a higher likelihood of responding to chemotherapy compared to BL2 and LAR subtypes [6]. In addition, LAR tumors are less sensitive to standard chemotherapy and may benefit from treatment with AR-targeted therapies [10]. Therefore, accurate identification of TNBC molecular subtypes is critical for selecting the most appropriate treatment for each patient.

The Precision Medicine Initiative was launched in late 2015, seeking an appreciated goal that involves personalizing medicine, starting from diagnostics to the treatment of patients in different disease states [11]. It utilizes many patient-related factors like lifestyle, environmental, and genetic factors, combining them into a precise patient-specific treatment plan model [12]. Precision oncology (PO) is a branch of precision medicine focused on cancer treatment. PO in the last few years has shown promising outcomes in promoting and extending disease-free survival (DFS) [13], particularly for TNBC.

Precision medicine divisions that are in use include diagnostic, stratifying, prognostic, and response divisions in which the functioning agents are the disease biomarkers [14]. Recent studies have identified potential therapeutic targets for each TNBC molecular subtype based on the affected pathways [15–18]. In addition, liquid biopsies, which involve the analysis of circulating tumor DNA and other biomarkers in the blood, show promise for non-invasive monitoring of TNBC and guiding treatment decisions [19].

In recent years, artificial intelligence (AI) has played a powerful role in precision oncology, offering the potential to improve biomarker discovery, patient stratification, precision diagnosis, and precision treatment by building models that learn from big data and extract hidden patterns to predict new knowledge from new data such as multi-omics datasets [20]. A recent study utilized AI to analyze gene expression data from TNBC patients, identifying a novel set of biomarkers with prognostic value [21].

Analyzing gene expression data from TNBC patients enables patient stratification into distinct responder groups with varying therapeutic outcomes, thereby aiding in decision-making about which patient groups are likely to benefit from specific therapies. Moreover, this reduces therapy costs by giving the right drug to the right patient [22,23]. AI can offer effective solutions by learning from large volumes of omics data [24]. Moreover, AI can assist in precision treatment by identifying potential therapeutic targets and predicting the response to treatment. A recent retrospective study of a cohort of TNBC patients confirmed that stromal tumor-infiltrating lymphocytes (sTILs) serve as robust biomarkers for therapeutic response following neoadjuvant chemotherapy [25,26]. AI can aid in discovering novel drug targets for cancer by analyzing extensive omics data, including genomics, proteomics, and protein–protein interaction networks [27,28]. For example, a tree-based classifier trained on protein–protein interactions, metabolic pathways, transcriptional interactions, as well as genomic and proteomic data, can predict morbid and druggable genes [27]. In another study, a support vector machine (SVM) classifier was built to classify proteins into drug targets and non-drug targets for breast and other cancers [28].

This study has a dual purpose, with the first objective being to apply AI to augment the ordinary differential expression analysis process to precisely identify the most relevant TNBC hub genes that can be used as biomarkers in diagnosis and prognosis and as targets for molecular modeling in new drug development. The second objective is to construct and build interpretable, high-accuracy AI models for use in precise diagnosis and precise patient stratification, which are two of the main aspects of precision oncology.

## 2. Materials and methods

### 2.1. Data wrangling

**Fig. 1:**
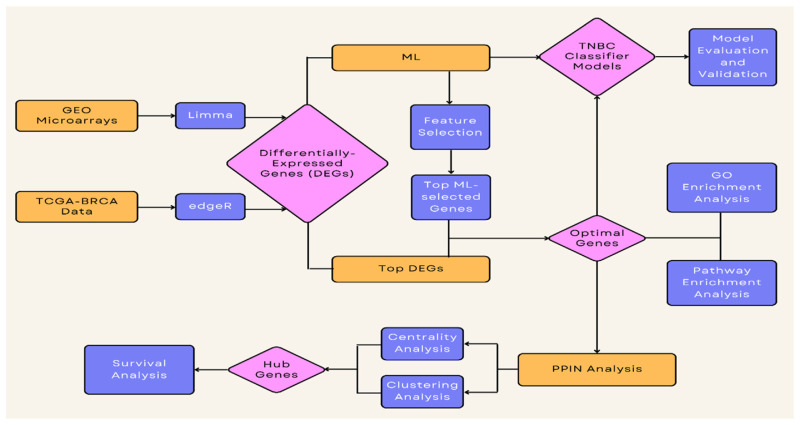
A high-level diagram representing a graphical abstract of the study workflow.

In this study, we focused on analysis of transcriptomic data to identify the differentially expressed genes between TNBC and non-TNBC cases. Transcriptomics technologies study the transcriptome, which comprises the sum of all of RNA transcripts in an organism. The expression of the information recorded in the genomic DNA is achieved through transcription to mRNA which is a transient intermediary molecule in the information network. A transcriptome is an in-time snapshot of the total transcripts present in a cell and this explains the rationale behind selecting this data type for analysis. Measuring the gene expression gives information on how genes are regulated in different cases and reveals their biological relevance. It can also help to predict the functions of unannotated genes from previous studies [29]. In the post-genome era, there have been many high throughput technologies that produce large amounts gene expression data. NCBI established the GEO repository in 2000 to host and publicly disseminate this data. The primary function of the GEO repository is to archive data and act as a central repository for data storage and retrieval. Currently, GEO is the largest fully open gene expression resource in the world. As of this writing, the GEO database contains more than 50,000 sample sets with approximately 1,000,000 individual expression measurements and 13,000,000 gene expression profiles for more than 100 organisms submitted by nearly 1,500 labs across a wide range of biological contexts such as disease, evolution, metabolism, toxicology and immunity, etc. The majority of the data is provided by research communities in accordance with journal qualifications requiring that microarray data be deposited in a publicly available repository, with the goal of allowing independent evaluation, reanalysis and full availability of all parts of a study [30]. GEO database mining techniques include screening for differential expression, molecular signaling, correlation, and gene regulation networks. The Cancer Genome Atlas (TCGA) is a large-scale database containing sequencing results, which provides comprehensive cancer genomic datasets on different tumor contexts including tumor staging, metastasis, molecular subtypes, survival, and clinical data for researchers [31]. In our study, we utilized data from the gene expression omnibus (GEO) repository of the NCBI as well as breast cancer data from TCGA. We used 6 RNA gene expression GEO microarrays, namely GSE7904, GSE21653, GSE43358, GSE45827, GSE65194, and GSE76275, for differential gene expression analysis to find the most relevant genes. All six datasets included in the study belong to the GPL570 platform. We included the microarray datasets according to these inclusion criteria: (i) the organism is *Homo sapiens*, (ii) Cases of TNBC and non-TNBC patients, (iii) primary tumors without treatment; (iv) all samples are RNA samples for transcriptome analysis, and (v) expression profiling by array is the study type used. First, we downloaded the data from the NCBI-GEO repository (Website URL: https://www.ncbi.nlm.nih.gov/geo/). The “.cel” files were read and further normalized using the robust multi-chip average (RMA) algorithm using the Affy R package [32]. This RMA normalization step ensures background correction and the removal of technical variation between arrays [32]. Feature data were cleaned by removing probes without Entrez gene identifiers. Probes with Entrez identifiers but missing gene symbols were annotated using the AnnotationDbi [33] and org.Hs.eg.db [34] R packages. The study workflow graphical abstract is represented in Fig. 1.

In parallel, we utilized the breast cancer data available at the cancer genome atlas (TCGA) database (Website URL: https://www.cancer.gov/ccg/research/genome-sequencing/tcga), denoted as TCGA-BRCA project which was downloaded from genomic data commons (GDC) portal (Website URL: https://portal.gdc.cancer.gov/projects/TCGA-BRCA) for a more precise and augmented analysis. The TCGAbiolinks R package [35] was used to gather the TCGA-BRCA project data. Transcriptomic profiling data was inquired and downloaded from the IlluminaHiSeq_ RNASeq platform. The downloaded data was used to construct a summarized experiment object in the presence of SummarizedExperiment R package [36]. The expression assay matrix data was extracted, normalized, and quantile-filtered. The sample data was used to identify the target groups to be analyzed.

### 2.2. Identification of target groups

The phenotype data for each dataset provides all the information about samples, including the clinical group to which each sample refers. The three groups identified in the overall collection of datasets were TNBC, non-TNBC, and healthy groups.

### 2.3. Differential expression analysis

For both GEO and TCGA data, samples representing healthy cases were dropped, leaving only the TNBC and non-TNBC samples and eventually directing us toward a binary classification problem. After the removal of the healthy samples, the GEO microarray gene expression data from each dataset was fed into the LIMMA algorithm in R [37]. A design matrix for each dataset was constructed based on the target groups of samples. Then, a contrast representing the differential between groups was made using the constructed design. We calculated array weights from gene expression data and our design matrix to overcome any problem that may occur if one or more samples showed outliers [38]. The array weights matrix was used in fitting the data with the LIMMA model. Consequently, the LIMMA-fitted data was then used to fit the design contrast built and, finally, to undergo the eBayes fit to compute the moderated statistics of differential gene expression [39]. The differential expression of each dataset reveals how many genes were downregulated, upregulated, or not changed in each specified contrast [37]. We used adjusted P.Value (adj.P.Value) and log fold change (logFC) as statistical criteria for gene selection. The adjusted p-value corrects for the multiple comparison problem, which happens when several statistical tests are performed simultaneously. It shows the probability that the observed variance in gene expression is due to chance, after accounting for repeated tests. Many adjustment methods can be used. However, Bonferroni correction and Benjamini-Hochberg methods are the most popular methods. Here, we set the adjustment method to Benjamini and Hochberg method which is a philosophically distinct and more powerful adjustment approach. Unlike the Bonferroni approach, which regulates the false positive rate, this method controls the false discovery rate. In other words, FDR represents the predicted fraction of false positives among all positives that rejected the null hypothesis rather than among all tests performed.


$$FDR=\frac{False\;Positives}{False\;Positives+True\;Positives}$$

The FDR technique ranks P values in ascending order and multiplies them by m/k, where k is the position of a P value in the sorted vector and m is the number of independent tests [40]. The log fold change is defined as the logarithm (base 2) of the ratio of gene expression levels between two conditions. Its objective is to quantify the magnitude of the expression change. A logFC of one signifies a doubling of expression, while −1 represents a halving [37]. The filters we applied to the infinite top table of the differential expression analysis result were an adj.P.Value less than 0.01, and a logFC greater than 1.5. The differentially expressed genes (DEGs) obtained after the application of the chosen criteria were cleaned from duplicate gene symbols, keeping only the most informative probes. For TCGA data, the differential gene expression analysis was achieved using the edgeR algorithm introduced via the TCGAbiolinks R package by introducing the target group filtered expression matrices. With setting analysis criteria equal to false discovery rate (FDR) less than 0.01 and logFC greater than 1.5, the significant differentially-expressed genes were obtained. The common genes shared between the GEO and TCGA data analyses were selected. After that, we moved in two directions: the first was to select the top DEGs from each dataset’s filtered DEGs, and the second was to merge the filtered DEGs from all six datasets.

### 2.4. AI-based feature selection

The unique merge of filtered DEGs with their retrieved gene expression data and target groups was intended to enter the ML workflow. To optimize ML model performance, it is important to have so many observations to allow more efficient model training and learning from the data, which in turn prevents the problem of overfitting, in which the model performs well on the seen experimental data but not on the real unseen data [41,42]. To achieve that, we extended samples with other GEO datasets, namely GSE83937, GSE95700, GSE103091, GSE135565, GSE157284, and GSE167213. These additional datasets were chosen according to the same selection criteria used to select the main datasets mentioned in Section 2.1. The extended data sample count became larger, which was better [41,42]. Thereafter, the data was fed into an ML workflow, starting with data preprocessing involving feature scaling and encoding the categorical target classes using the Scikit-learn Python library [43]. Various feature selection techniques, including embedded, filter, and wrapper algorithms, were applied to select the most relevant probes [44]. The main difference between these methods is that filter methods is correlation-based measuring the relation of the features to the dependent variable, but embedded and wrapper methods are model-based measuring the feature importances after model training. We used a random forest classifier (RF) [43], recursive feature elimination with 5-fold cross-validation (RFECV) [43], mutual information classifier (MIC) [43], and minimal redundancy maximal relevance (MRMR) [45] algorithms for the feature selection step.

#### 2.4.1. Embedded feature selection algorithm

Feature selection using embedded methods is a result of the model training process, thereby calculating the importance of each feature. Embedded methods evaluate the interaction of features with the constructed model. The training step is passed only once, making these methods faster than wrapper methods and having fewer computational requirements. Also, they are encountered with higher accuracy and less susceptibility to overfitting. Once the model is trained, the feature importance is obtained, and the features with low importance can be removed to finally have a list of the most important and interacting features. Embedded methods include regularization methods like Lasso (L1), Ridge (L2), and ElasticNet (L1/L2), as well as tree-based algorithms such as ExtraTree and random forest. The Random Forest Classifier (RFC) is the embedded method that was implemented in this study.

The RFC classifier is an ensemble ML algorithm from the Scikit-learn library that is considered one of the embedded methods of feature selection. It is characterized by low computational power, high speed, a high degree of interpretability, high performance, and low overfitting [46,47]. A random forest consists of decision trees built over a random extraction of the observations and features from the dataset. Not every tree sees all the features or all the observations, and this guarantees that the trees are de-correlated and therefore less prone to overfitting. In our classification problem, the RFC algorithm measures the impurity by the Gini index.


$$\text{Gini Index}=1-\sum_{i=1}^{n}{\left(P_{i}\right)}^{2}$$

Where *P*_*i*_ is the predicted class probability. The more the feature decreases the impurity, the more important the feature [41].

#### 2.4.2. Wrapper feature selection algorithm

These methods are model-based and select features according to the feature importance metric calculated when training an estimator model on the data. Therefore, they select the highest-performing feature set for the used estimator model, resulting in a higher model prediction power. Recursive feature elimination (RFE) is a wrapper feature ranking technique in which the base estimator model is trained and the least ranked feature is removed, then the process is recursively repeated until the required number of features is finally reached [43]. RFECV has a built-in cross-validation algorithm to validate the selected features. It eliminates the multi-colinearity between features, therefore preventing overfitting [43,48]. Recursive Feature Elimination with Cross-Validation RFECV has a built-in cross-validation algorithm to validate the selected features. It eliminates the multi-colinearity between features, therefore preventing overfitting [43,48]. We used RFECV with a random forest classifier as a base estimator and noticed that RFECV is computationally expensive and takes more time than other algorithms.

#### 2.4.3. Filter feature selection algorithms

##### 2.4.3.1. Mutual information classifier (MIC)

MIC is a univariate mutual information-based feature selection method that measures the mutual information gain between each feature and the target variable by the difference between the entropy of the feature and the conditional posterior entropy of the feature given the target variable. The lowest conditional entropy feature is the most important, having the most mutual information gain [43,49]. Entropy is the fundamental unit of information of a random variable. The entropy is the uncertainty measure and quantifies the amount of shared information and the uncertainty of the probability distribution of a random variable X as,


$$H(X)=-\sum_{x\in X}p(x)\;log\;p(x)$$

Where:

- *x* is any possible event or value that belongs to the X vector space with an index *i*.
- *p*(*x*) is the probability distribution of the event *x*.

Conditional entropy can be estimated given other events. So, for a random variable *X*, the conditional entropy or the amount of uncertainty given Y can be measured using the formula:


$$H(Y)=-\sum_{y\in Y}p(y)\sum_{x\in X}p(y)logp(x|y)$$

Where:

- *p*(*x*|*y*) is the posterior probability of an event *x* given *y*

The Mutual Information between X and Y is the amount of information shared by X and Y, and can be calculated as follows:


I(X;Y)=H(X)-H(X∣Y)

This is the difference between two entropies—the uncertainty before Y is known, H(X), and the uncertainty after Y is known, H (X|Y) which represents the probability of X given Y.

The mutual information is symmetric, that is,


I(X;Y)=I(Y;X)

and is zero if and only if the variables are statistically independent [43].

##### 2.4.3.2. Minimal redundancy maximal relevance (MRMR)

The MRMR algorithm aims to iteratively select features having a maximal correlation with the target variable and a minimal correlation with each other [43,50]. The selected features from all four algorithms are ranked based on their presence percentage. We selected the highest-ranked features from those obtained by all four models. Then, we selected probes that were common with LIMMA-selected top probes. MRMR is a multivariate filter method based on mutual information. If we want to select an optimal feature set S with maximal relevance V to the target variable Y, and minimal redundancy W between features. Then we have


$$\begin{array}{l} V=\frac{1}{|S|}\sum_{x\in S}I(x;Y)\\ W=\frac{1}{|S{|}^{2}}\sum_{x_{i}x_{j}\in S}I(x_{i},x_{j})\\ MID=\text{max }(V-W) \end{array}$$

Where *MID* is the mutual information difference, which represents the MRMR evaluation criterion [45].

### 2.5. AI classifier model building

Our case is a binary classification problem. We applied several ML models to the final data, namely Random Forest Classifier (RFC) [43], Support Vector Machine Classifier (SVC) [43], Gradient Boosting Classifier (GBC) [43], HistGradientBoosting Classifier (HGBC) [43], Extreme Gradient Boosting Classifier (XGBC) [51–53], CatBoost Classifier (CTBC) [51,53], AdaBoost Classifier (ADBC) [51,54], and Multi-Layer Perceptron Classifier (MLP) [43,51]. Each model was subjected to a hyperparameter optimization algorithm named Optuna that performs several suggested trials over a range of hyperparameter values and eventually selects the best values according to the tested model performance metrics specified [55].

### 2.6. Model validation

The tuned models were trained, tested, and evaluated for performance and further validated using unseen GEO dataset GSE61724.

### 2.7. GO and Reactome enrichment analysis

Gene ontology (GO) and pathway enrichment analysis for the selected genes were achieved using the R packages ClusterProfiler and ReactomePA, respectively [56–60]. For GO enrichment analysis, we used “org.Hs.eg.db” as the annotation database [34], searched for all the GO categories with false discovery rate (FDR) as the control metric, and set the p-value cutoff to 0.01 in the “enrichGO” function of the ClusterProfiler package. For pathway enrichment analysis, we used the “enrichPathway” function of the Reactome PA package with parameter values of 0.05 as the p-value cutoff and FDR as the control.

### 2.8. Protein–protein interaction (PPI) network analysis

We used STRING (Search Tool for Retrieval of Interacting Genes) database version 11.5 (Website URL: https://string-db.org/) to construct a PPI network from the selected top genes [61]. Then, we transferred the STRING-constructed network to the Cytoscape tool version 3.10.0 (Website URL: https://cytoscape.org/) for visualization [62]. We performed clustering and module analysis by using a Cytoscape application called MCODE (Molecular Complex Detection) version 2.0 [63]. The hub genes were identified and detected using the Cytohubba plugin (version 0.1) [63].

### 2.9. Survival analysis of hub genes

We used the Kaplan–Meier plotter web-based tool (Website URL: https://kmplot.com/) to measure the correlation between the expression of the hub genes and survival in breast tumor samples to validate their role as biomarkers [63,64]. This tool implements statistical algorithms such as Cox proportional hazards regression and false discovery rate [64,65].

## 3. Results

### 3.1. Identification of differentially-expressed genes

The data wrangling step involved gathering gene expression, features, and phenotypic data from six GEO microarray datasets. Gene expression data were normalized and background-corrected using the RMA algorithm. Features were cleaned from probes that have no Entrez gene identifier, reducing the number of features. After dropping out healthy samples, a design matrix and contrast were made based on the target groups of samples. The fitted contrast summarized the counts of up-regulated, down-regulated, and non-changed genes, as shown in Table 1. The differentially expressed genes were filtered by an adj.P.Value < 0.01, and a logFC >1.5 giving rise to a reduced table arranged with respect to adj.P.Value in ascending order.

**Fig. 2:**
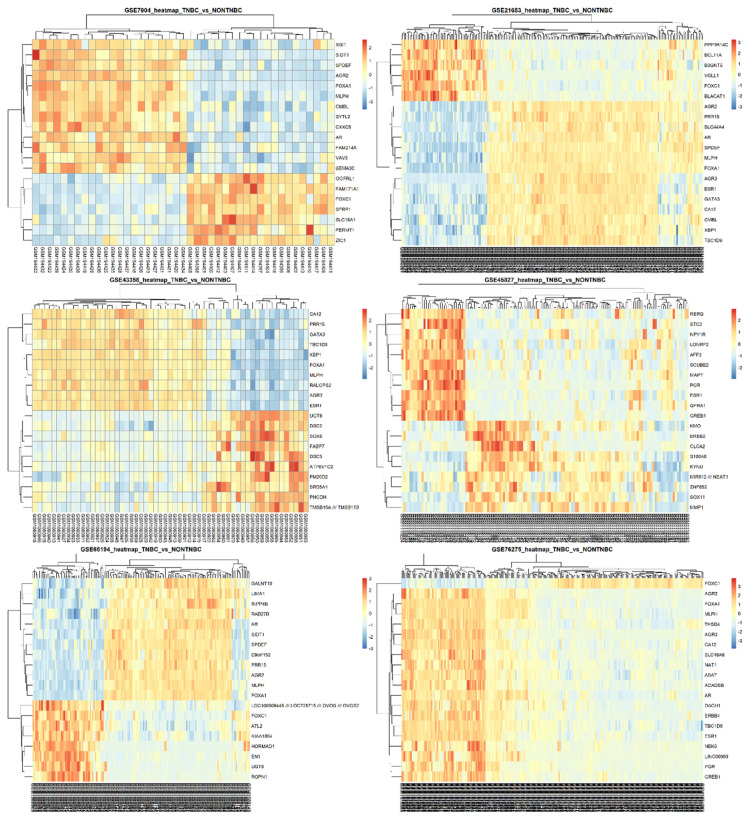
Heatmaps of the top 20 DEGs selected from GSE7904, GSE21653, GSE43358, GSE45827, GSE65194, and GSE76275 datasets. Cluster colors show a high separation of the 2 clusters (TNBC and non-TNBC), indicating the relevance of the selected genes.

**Fig. 3:**
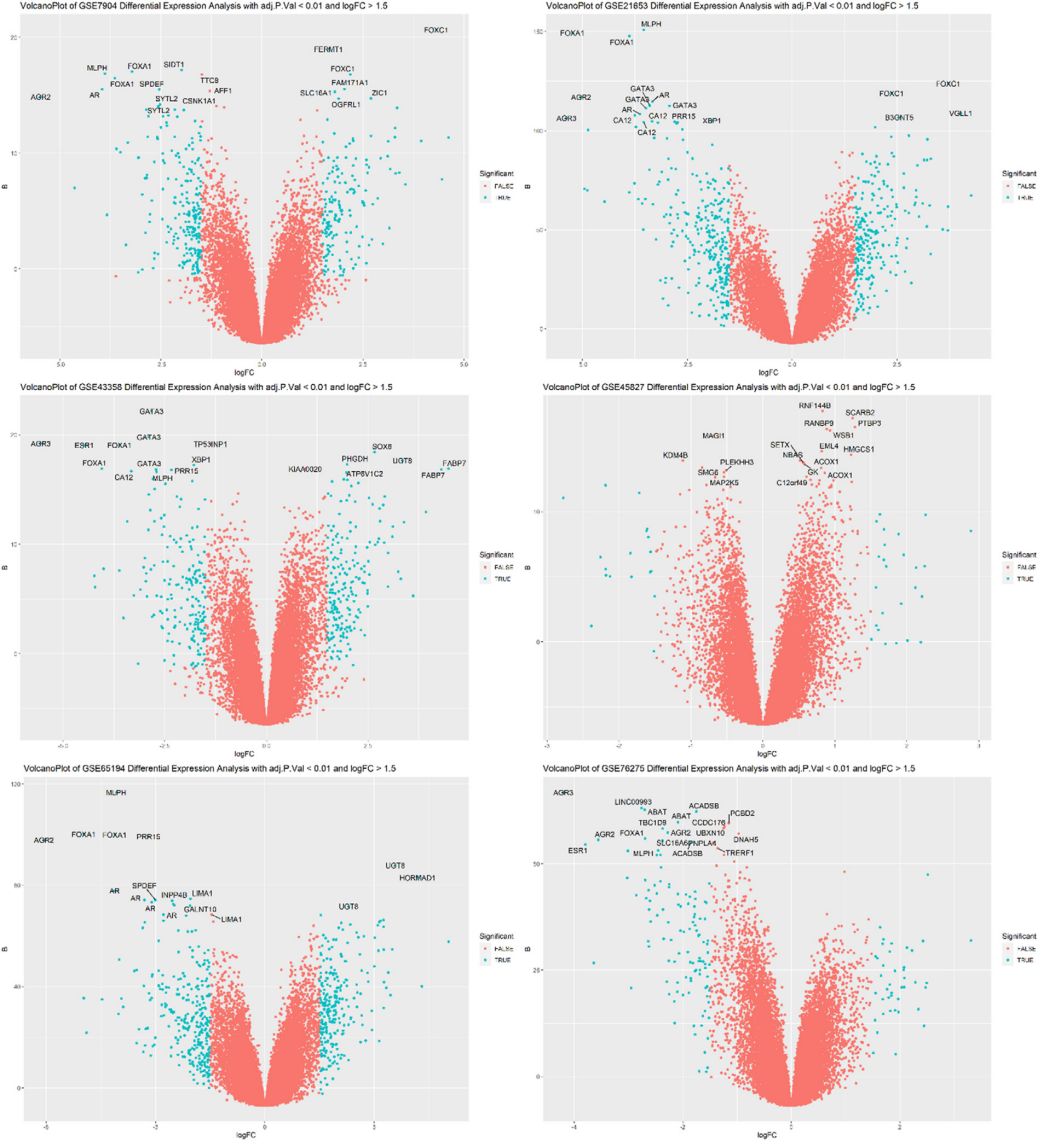
VolcanoPlots of the top 20 DEGs selected from GSE7904, GSE21653, GSE43358, GSE45827, GSE65194, and GSE76275 datasets. Gene symbols represent the selected genes.

**Fig. 4:**
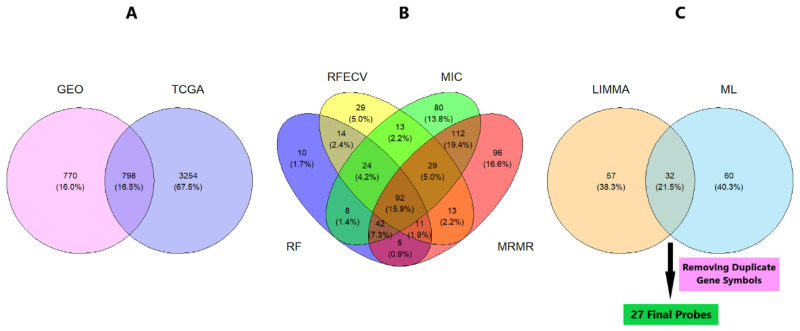
Venn diagrams showing the intersections between A) Significant DEGs obtained from GEO and TCGA datasets, B) Probe features selected from 4 ML models, and C) Final DEGs obtained from LIMMA algorithm and ML feature selection algorithms.

Some probes were noticed to refer to more than one gene symbol and were therefore considered non-informative, so it was better to remove them by dropping duplicate gene symbols from filtered DEGs. We did not remove the duplicate gene symbols in the earlier steps due to the fact that, before the filtration step, the differential strength of each probe was still unknown. After applying filters, the removal of duplicate gene symbols and their corresponding probes could eventually preserve the most informative DEGs. We decided to select the top 20 DEGs from each dataset that are the most significant and informative genes. Heatmaps of the top 20 DEGs selected from each dataset are shown in Fig. 2, and their volcano plots are shown in Fig. 3. Merging the top 20 DEGs of all six GEO datasets and the top 20 DEGs from TCGA dataset resulted in 140 DEGs with only 89 unique genes. The unique filtered DEGs of all six datasets were merged into 1568 genes, giving rise to an ultimate unique total of 798 genes. In parallel, the edgeR pathway in TCGA analysis resulted in 4052 significant genes of which there are 1743 upregulated and 2309 downregulated genes. The unique 798 genes obtained from GEO data LIMMA analysis were common in TCGA significant DEGs as well, and a Venn diagram shows this intersection in Fig. 4A. Therefore, these unique DEGs were intended to be fed into the ML workflow for classifier building. At this step, the number of samples in the merged data was 915 from GEO and 1059 TCGA samples forming a total of 1974 samples, which was quite small and not expected to train a highly-performant model. To increase the number of observations, we extended samples with samples from GSE83937, GSE95700, GSE103091, GSE135565, GSE157284, and GSE167213 datasets. The extended data sample count became 2690 samples of 1315 TNBC and 1375 non-TNBC samples. These combines 2690 samples from both GEO and TCGA was used to train the ML models.

### 3.2. AI-based feature selection

We started preprocessing the data by applying the feature scaling algorithm. Here we chose the standard scaler method from the Scikit-learn Python library and label-encoded the target classes.

Four ML models were applied for the feature selection step, including RFC, RFECV, mutual information classifier, and MRMR. The number of features selected by each algorithm was 206 by RFC, 225 by RFECV, 400 by MRMR, and 400 by MIC, as summarized in Table 2.

**Fig. 5:**
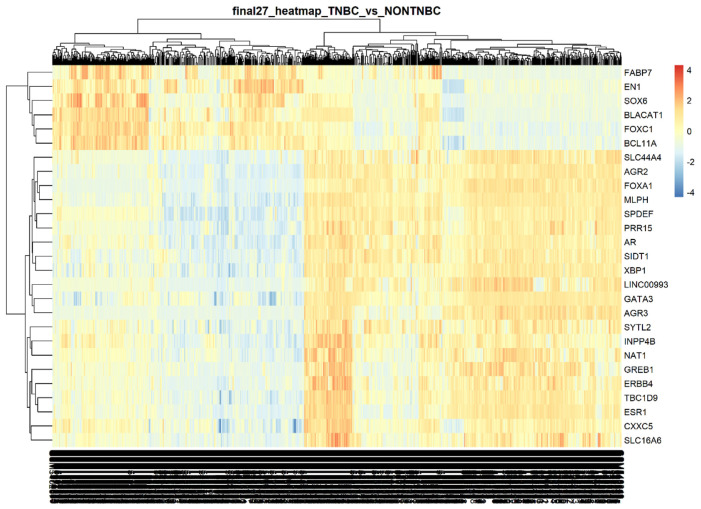
Heatmap showing the clustering of the top 27 genes resulting from all 6 GEO datasets and TCGA data analysis.

Then, we constructed an extended list of all probes selected by the 4 models, of which the length is the 1231 probes selected. The features were ranked by the number of models from which they were selected. The highest-ranked features that were selected by all 4 models were 92 probes representing the most relevant genes selected by ML models as shown in the Venn diagram in Fig. 4B. After that, we intersected the top LIMMA-ranked probes and the top model-ranked probes to get the probes that were common and selected by both the LIMMA and model ranking systems. One probe having multiple Entrez identifiers was removed as it was non-informative, and duplicate gene symbols were removed (Fig. 4C). Eventually, 27 probes representing the topmost relevant features in our data were selected and represented in Table 3. A heat-map clustering of the 27 genes’ expression matrix is shown in Fig. 5.

### 3.3. AI classifier model building

Several ML classifiers were chosen for the binary classification task. To get the best performance from each model, we fine-tuned each model’s hyperparameters using a powerful Python library named Optuna. For each model, and after several trials involving a predefined range of hyperparameter values, the Optuna algorithm selected the best trial with the best hyperparameters and leveraged the highest values of the model’s performance metrics used in the experiment. Then the optimized models were trained, and evaluated with several classification performance metrics, including accuracy, precision, recall, F1-score, and area under curve AUC [66].

### 3.4. Model validation

For model validation, we utilized the unseen data from the GSE61724 dataset from the GEO repository. The validation scores of the models reflects their interpretability and reliability in differentiation between TNBC and non-TNBC breast cancer cases. The aforementioned classifiers showed high area under receiver operating characteristic curve ROC-AUC values, with the highest scores from CTBC, XGBC, RFC, and MLP.

**Accuracy** calculates the proportion of the total number of predictions that were correct. It is the number of correct predictions divided by the total number of predictions [66].


$$\begin{array}{l} Accuracy=\frac{Number\;of\;correct\;predictions}{Total\;number\;of\;predictions}\\ =\frac{TP+TN}{TP+FP+TN+FN} \end{array}$$

**Precision** shows what proportion out of all positive predictions was correct. To calculate it, you divide the number of correct positive results (TP) by the total number of all positive results (TP + FP) predicted by the classifier. Precision does well in cases when you need to or can avoid False Negatives but can’t ignore False Positives [66].


$$Precision=\frac{Number\;of\;correct\;positives}{Total\;number\;of\;positives}=\frac{TP}{TP+FP}$$

**Recall** shows a proportion of correct positive predictions out of all positives a model could have made. To calculate it, you divide all True Positives by the sum of all True Positives and False Negatives [66].


$$Recall=\frac{Number\;of\;correct\;positives}{Number\;of\;all\;positives}=\frac{TP}{TP+FN}$$

The **F1 Score** tries to find the balance between precision and recall by calculating their harmonic mean. It is a measure of a test’s accuracy where the highest possible value is 1. This indicates perfect precision and recall [66].


$$\begin{array}{l} F1\;score=Harmonic\;mean\;of\;precision\;and\;recall\\ =2\times \frac{Precision\times Recall}{Precision+Recall}\\ =\frac{2TP}{2TP+FP+FN} \end{array}$$

**Fig. 6:**
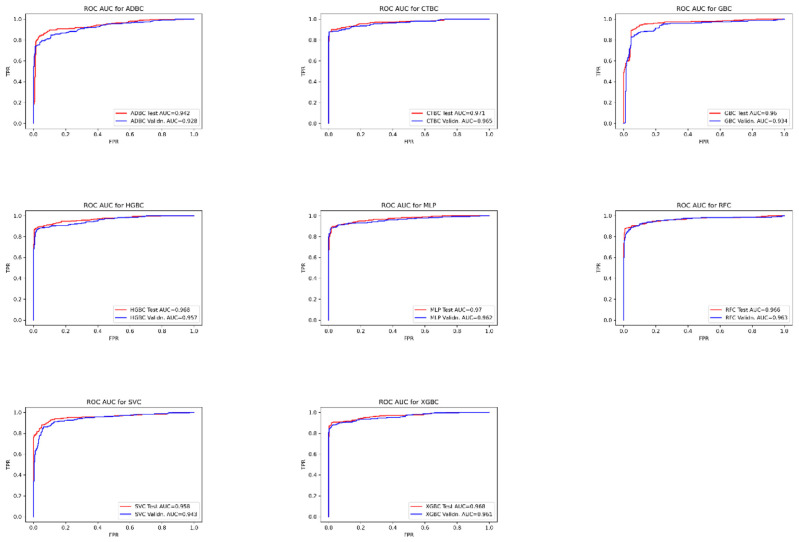
ROC AUC curves for model testing and validation.

**A Receiver Operating Characteristic curve** or **ROC curve** is created by plotting the cumulative distribution function of true positive rate (TPR) against the false positive (FPR). The area under the ROC curve (ROC AUC) is used for evaluating the performance. The higher the AUC, the better the performance of the classifier model trained [66]. The maximal possible percentages of accuracy, precision, recall, F1, and ROC AUC scores for all tuned models in both test and validation stages were summarized in Table 4. The area under the ROC curves for each classifier in both testing and validation are represented in Fig. 6.

### 3.5. GO and Reactome enrichment analysis

By looking for all GO categories through the ClusterProfiler package, we obtained a GO enrichment result involving only biological processes (BP) and molecular functions (MF) with no cellular component (CC) enrichment. The most enriched BP terms included gland development, cell fate commitment, and miRNA transcription. The most enriched MF terms included transcription coactivator binding, dystroglycan binding, steroid binding, and DNA-binding transcription activator activity.

**Fig. 7:**
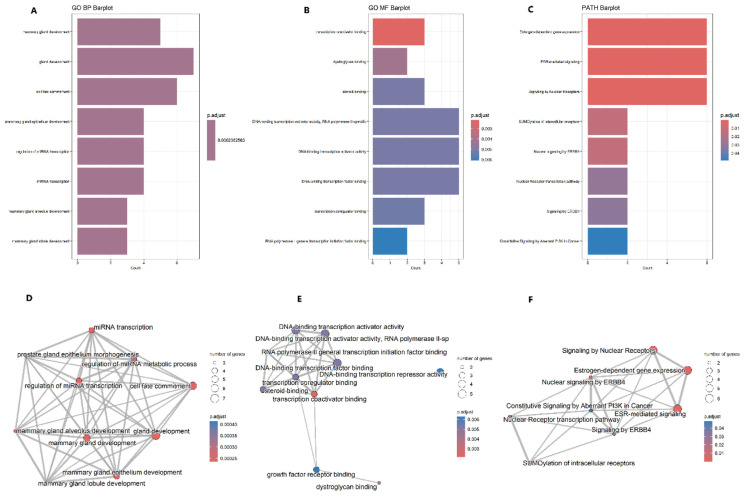
The most enriched GO biological processes (BP) and molecular functions (MF) as well as Reactome pathways for the top 27 genes represented in bar plots A, B, and C respectively, and enrichment maps D, E, and F respectively.

Reactome pathways that were mostly enriched are estrogen-dependent gene expression, ESR-mediated signaling, and signaling by nuclear receptors. Bar plots and enrichment maps of the most enriched GO BP and MF terms as well as Reactome pathways are represented in Fig. 7.

### 3.6. Protein–protein interaction (PPI) network analysis

**Fig. 8:**
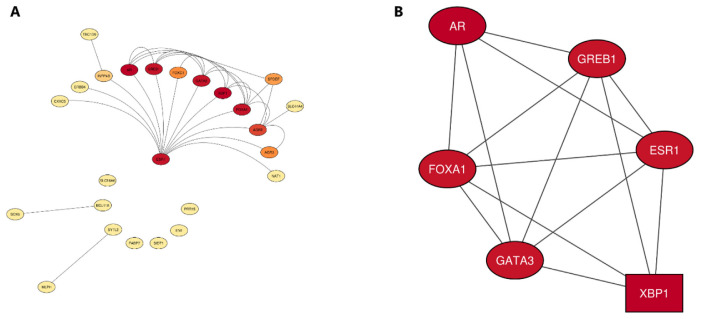
A) PPI network of the 25 top mapped genes (STRING database view). B) MCODE-identified cluster showing XBP1 as a seed.

Using the STRING database, we constructed a PPI network from the selected top 27 genes. From those 27 genes, only 25 were mapped. The non-mapped genes were BLACAT1 and LINC00993. The resulting PPI network showed 25 nodes, 32 edges, an average node degree of 2.56, and an average local clustering coefficient of 0.635. The PPI significantly revealed a high degree of interactivity, with a PPI enrichment p-value of less than 1.0e–16. The high interactivity of the PPI provided us with evidence that the proteins are biologically connected (Fig. 8A). Clustering and module analysis via the MCODE plugin yielded only one cluster with a score equal to 5.6 using a degree cutoff of 2, a node density cutoff of 0.1, a node score cutoff of 0.2, a K-Core of 2, and a maximal depth of 100 as clustering parameters. The resulting cluster was structured with 6 nodes and 14 edges. The 6 nodes were represented by ESR1, FOXA1, GATA3, XBP1, GREB1, and AR genes, with XBP1 being the seed of the cluster (Fig. 8B). Moreover, the Cytohubba plugin helped identify 7 downregulated hub genes by intersecting the results of the 3 most important metrics: mean clique centrality (MCC), node degree, and closeness scores. Six of the discovered hub genes were intramodular, structuring the skeleton of the MCODE-discovered cluster, and the seventh one was AGR2, which was the only one to be an extramodular hub gene, as shown in Table 5.

### 3.7. Survival analysis of the hub genes

**Fig. 9:**
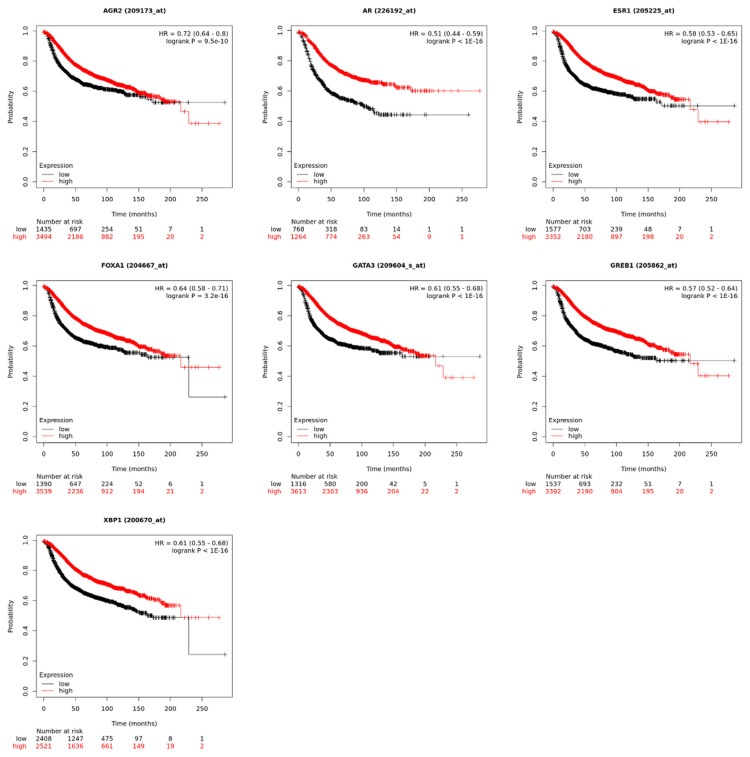
Kaplan–Meier plots of the 7 hub genes survival analysis.

Survival analysis of the hub genes revealed that high expression of ESR1, GATA3, XBP1, GREB1, AR, and AGR2 and low expression of FOXA1 contributed to extended disease-free survival (DFS). The result provided a piece of evidence that the hub genes can be considered reliable prognostic biomarkers (Fig. 9).

## 4. Discussion

In this study, our objective was to identify hub genes specific to TNBC and to build an AI classifier model to help precision medicine, especially in diagnostics and patient stratification. We worked on TCGA-BRCA data and 6 GEO microarray datasets, namely GSE7904, GSE21653, GSE43358, GSE45827, GSE65194, and GSE76275. After RMA-normalization and background correction, we identified 798 unique DEGs filtered from all 6 datasets using the LIMMA R algorithm that were also common with TCGA edgeR-selected data. Also, we selected the top 20 DEGS from each dataset to have a total of 140 DEGs, represented by 89 unique DEGs. The training data was constructed for the 798 genes from both GEO and TCGA data. We extended the sample count by including samples from the GEO datasets GSE83937, GSE95700, GSE103091, GSE135565, GSE157284, and GSE167213. Then, we introduced the transposed expression matrix of the 798 DEGs to an ML-based feature selection workflow by applying 4 ML algorithms, including RF, RFECV, MRMR, and MIC, to finally have the top 92 DEGs. After that, we intersected the common genes between the ML-selected 92 DEGs and the top LIMMA-selected 89 DEGs to eventually get 27 genes that were considered the top most informative genes in the case. The selected 27 genes were used to train 8 AI classifier models, which are RFC, SVC, XGBC, HGBC, CTBC, GBC, ADBC, and MLP. We optimized the model hyperparameters using the Optuna Python algorithm, which finally enhanced the model performance scores, with the best scores recorded with CTBC, XGBC, RFC, and MLP of which performances were also reported in previous studies [51,67,68]. The GO enrichment analysis of the 27 genes revealed gland development, cell fate commitment, and regulation of miRNA transcription as the most enriched BP terms. Gland development in TNBC is not driven by hormonal or Her2 signaling resulting in cancer cells with basal-like characteristics of normal breast epithelium leading to the limited responsiveness to the targeted therapies. Cell fate commitment is the process by which the undifferentiated cells decide to follow a specific developmental pathway, resulting in formation of distinct cell types with specialized functions. In the context of cancer, disruptions in cell fate commitment can contribute to tumorigenesis, progression, metastasis, and therapeutic resistance. In TNBC, disruption of cell fate commitment is a cause of heterogeneity and plasticity, increased differentiation to cancer stem cells (CSCs) that contribute to tumorigenesis and metastasis, and finally the activation of epithelial–mesenchymal transition (EMT) pathways which results in increased invasiveness [69]. Regulation of miRNA transcription promotes proliferation, invasiveness, and metastasis via regulation of EMT-related genes such as miR-21 upregulation which regulates PTEN expression [70]. The most enriched MF terms included DNA-binding transcription activator activity, steroid binding, and steroid coregulator binding. DNA-binding transcription activator activity as an enriched MF is about transcription factors that regulate genes by DNA binding. Some of these transcription factors when activated lead to progression and metastasis of TNBC, such as STAT3 which acts as a signal transducer and transcription activator [71]. Due to the independency on ER, PR, and Her2 hormonal receptor expression, TNBC is more aggressive and resistant to the targeted therapies due to relation to other steroid hormones such as androgens and glucocorticoids via binding to their receptors [72]. Steroid coregulator binding play a critical role in mediating the effects of androgen receptor signaling in TNBC cells leading to increased heterogeneity of TNBC and presence of subtypes that not express androgen receptor and resist the targeted treatments [72]. The Reactome pathway analysis resulted in the top enriched pathways, including estrogen-dependent gene expression, ESR-mediated signaling, signaling by nuclear receptors, nuclear signaling by ERBB4, and SUMOylation of intracellular receptors. Estrogen-related pathways including estrogen-dependent gene expression and ESR-mediated signaling have their impact depending on the fact that although TNBC cells do not express ERα, but are responsive to estrogen via ERα-independent pathways such as estrogen receptor Erβ, G protein-coupled estrogen receptor 1 (GPER-1), and the constitutively active estrogen-related receptors (ERRs). Brain metastatic colonization of TNBC cells promoted by estrogen is previously reported [73]. Ovariectomy decreased the frequency of brain metastases by 56%, and combined ovariectomy with the aromatase inhibitor letrozole further reduced the large lesion frequency to 14.4% of the estrogen control group. Moreover, it was demonstrated that increasing levels of circulating estrogens was sufficient to promote the formation and progression of ERα-negative cancers including TNBC [73]. The nuclear receptor (NR) superfamily contains hormone-inducible transcription factors that regulate gene expression. NR4A1 is reported to be downregulated in TNBC and restoration of NR4A1 expression inhibits TNBC growth and metastasis, suggesting that NR4A1 is a tumor suppressor in TNBC [74]. Furthermore, it was demonstrated that nuclear epidermal growth factor receptor nEGFR enhances resistance of TNBC to anti-EGFR therapies and can be considered as a functional molecular target in TNBC [75]. Differential expression analysis of the HER family genes in TNBC reported that increased expression of ERBB4 was associated with poor prognosis according to survival analysis [76]. SUMOylation of intracellular receptors is a posttranslational modification (PTM) involving the upregulation of small ubiquitin-like modifier (SUMO) in various cancers and plays a major role in tumor development. The dysregulation of SUMOylation mechanism can lead to cancer cell protection from stresses exerted by either external or internal stimuli [77]. Thereafter, we constructed a PPI network on the STRING platform from the 27 genes, mapping only 25 genes. Then, the PPI network was visualized in Cytoscape, with only one cluster of 6 genes in the MCODE analysis. The cluster genes were found to be from the 7 hub genes identified in the CytoHubba plugin ranking analysis using the top 3 most important node ranking metrics, which were node degree, mean clique centrality, and closeness. The 7 hub genes identified were ESR1, FOXA1, GATA3, XBP1, GREB1, AR, and AGR2. A previous study that integratively analyzed differential gene expression on GEO dataset GSE76275 revealed that six genes namely ESR1, FOXA1, GATA3, GREB1, AR, and AGR2 are downregulated genes that allow progression of TNBC, of which ESR1, FOXA1, GATA3, and AR were identified as transcription factor genes [78]. Coexpression analysis showed that downregulated FOXA1, AGR2, and GATA3 genes can serve as hub nodes. Functional annotation revealed their enrichment in GO keywords such as negative control of cell proliferation, negative regulation of epithelial to mesenchymal transition, and positive regulation of cadherin-mediated cell adhesion. Thereby, their essential involvement in TN-specific traits such as metastasis and rapid proliferation was highlighted [78].

The majority of existing reports on FOXA1 have found that downregulation is associated with poor prognosis. For example, FOXA1 has been identified as an EMT regulator. Its expression was found to be positively associated with disease-free survival rates in ER-positive breast tumors. A surprising and intriguing observation about FOXA1 is a favorable relationship between hypermethylation and parity. It was found that FOXA1 regulates ERα expression and suppresses the basal phenotype, suggesting a relationship between reproductive exposures and ER-negative breast tumors [78]. In a real-time qPCR study on breast cancer cell lines from American type tissue culture collection, FOXA1 was reported as an independent subtyping biomarker for TNBCs identification, and halts the triple negative feature of cancer cells by suppressing *SOD2* expression that helps cells bypass apoptosis and inhibiting *IL6* that enables stem-like features and invasive nature of TNBC [79].

GATA3 has been shown to regulate cell adhesion-related genes, which modulates triple-negative cell metastasis [78]. Analysis of downregulated ESR1 functional enrichment revealed that it has a negative effect on apoptosis leading to the progression of TNBC. All evidence suggests that ESR1, a downregulated gene, is a viable target in antitumor protein treatment of TNBC breast tumors, due to its effects and protein interactions [78]. The *ESR1* is highly polymorphic, and Single nucleotide polymorphisms (SNP) in four *ESR1* gene variants (rs2234693, rs9340799, rs3020314, rs3798577) and six *ESR2* gene variants (rs928554, rs944459, rs4986938, rs1256049, rs1256030, rs1271572) were reported to be linked with altered risk of TNBC in 488 BC patients comprising 130 TNBC and 358 non-TNBC patients in a study performed by real time qPCR [80].

In TNBC, XBP1 is usually upregulated and linked to tumor development and a poor prognosis. However, our analysis revealed XBP1 downregulation as augmented by other studies [81]. Downregulation of XBP1 can be linked to regulatory actions of certain microRNAs during endoplasmic reticulum stress and the unfolded protein response [82].

A previous study reported AR downregulation in TNBC [78], and different subtypes of TNBC with different AR status were identified in another study [83]. AR-negative TNBC was reported in 361 patients of 487 and comprised mainly of ductal carcinoma with higher grade and more lymphocytic infiltration and central fibrosis than AR-positive cases. Moreover, coexpression of AR and FOXA1 was seen with different status that are AR+/FOXA1+, AR+/FOXA1−, AR−/FOXA1+, and AR−/FOXA1−. In AR−/FOXA1-cases, the pathological type is mainly ductal carcinoma associated with more central fibrosis and higher grade of ductal carcinoma *In situ* [83].

AGR2 expression was significantly associated with histologic type, histological grade, estrogen status and progesterone status [78]. In this study, we reported downregulation of AGR2 in TNBC which is augmented with previous studies [78,84]. The AGR2 gene is significantly downregulated in TNBC and strongly correlated with FOXA1 due to its direct regulation by Erα [78].

We reported GREB1 downregulation consistently with previous studies [78]. Other studies stated that GREB1 is the most estrogen-interacting gene is GREB1, which is considered a potential clinical biomarker with no known function. GREB1 is a chromatin-bound ER coactivator and is responsible for ER-mediated transcription. GREB1-ER interactions in nearly half of ER^+^ primary breast cancers (non-TNBC) [85].

The impact of the selected hub genes was prominent in the GO and Reactome enrichment results. Survival analysis of the 7 hub genes showed extended DFS with variable degrees that were highest in the cases of ESR1, GATA3, AGR2, GREB1, and AR, providing evidence that these 5 genes can be used as consistent prognostic biomarkers. All of these hub genes were confirmed in other studies [78–85].

## 5. Clinical implications

While the current study focuses largely on the identification and characterization of a novel gene cluster linked with TNBC, we feel our findings have substantial clinical implications. Because of its aggressive nature and limited treatment choices, TNBC requires an accurate and early diagnosis. Our ML models show promising results in differentiating TNBC from non-TNBC instances. These models could be effective diagnostic tools, allowing clinicians to make more informed treatment decisions. Furthermore, the discovered gene cluster has the potential to form a prognostic and predictive biomarker panel. Understanding the expression patterns of these genes may allow us to stratify TNBC patients into subgroups with different clinical outcomes and therapy responses. This information can be used to guide individualized treatment methods, such as targeted medicines or appropriate chemotherapy regimens. We recognize that additional validation and prospective clinical investigations are required to properly grasp the clinical significance of our findings. The results provided here provide a good foundation for future translational research to improve patient outcomes in TNBC.

## 6. Limitations of the study

Despite the great results of this study, there are still the following limitations:

- **Limited size of the datasets:** The volume of data used to develop and evaluate the AI classifier models for TNBC diagnosis is one of the study’s limitations. Although the TCGA and GEO repositories are excellent sources of gene expression data, the dataset size may not allow producing the most reliable and broadly applicable AI models. Making use of a larger dataset may help the models become more generalizable to new data and increase the accuracy of precision diagnosis across a wider range of patient populations. This limitation might be addressed by existing clinical trials or future research that incorporates data from other sources.
- **The continued mutation and intra-tumoral heterogeneity**: As previously mentioned, TNBC in particular exhibits a significant degree of heterogeneity because of the activation of secondary pathways [6]. Because of this, there are fewer patients in each molecular subtype, which reduces the size of the datasets including gene expression and hence limits the interpretability of the ML classification prediction models that are built.

## 7. Conclusion

In our study, we demonstrated the great impact of ML-based feature selection in the journey of hub gene identification and ML classifier model building. Several ML classifier models were built, with CTBC, XGBC, RFC, and MLP showing the highest accuracy scores and being effective in the precision diagnosis of TNBC and patient stratification for optimal therapies. We identified 7 highly-interconnected hub genes in one cluster (ESR1, FOXA1, GATA3, XBP1, GREB1, AR, and AGR2) that can be used as robust prognostic biomarkers. The study results may be helpful in a deeper dive into TNBC and the discovery of new drug targets.

## Figures and Tables

**Table 1:** Count summary of overall upregulated, downregulated genes.

Dataset	Downregulated	Upregulated
GSE7904	2911	2944
GSE21653	10121	12355
GSE43358	4323	5011
GSE45827	4037	4286
GSE65194	10973	10634
GSE76275	8035	12248
TCGA-BRCA	2309	1743

**Table 2:** Number of features selected by each model.

Model	Selected Features Count
RFC	206
RFECV	225
MRMR	400
MIC	400

**Table 3:** The final top selected genes shared between LIMMA top genes and machine learning feature selection applied on the 798 common genes between TCGA-edgeR and GEO-LIMMA significant DEGs.

Probe ID	Gene symboL	Entrez gene ID
219734_at	SIDT1	54847
218211_s_at	MLPH	79083
220192_x_at	SPDEF	25803
225496_s_at	SYTL2	54843
224516_s_at	CXXC5	51523
212956_at	TBC1D9	23158
205030_at	FABP7	2173
200670_at	XBP1	7494
220559_at	EN1	2019
226961_at	PRR15	222171
209604_s_at	GATA3	2625
228241_at	AGR3	155465
235046_at	INPP4B	8821
237339_at	LINC00993	1.02E+08
205597_at	SLC44A4	80736
205225_at	ESR1	2099
214440_at	NAT1	9
205862_at	GREB1	9687
230748_at	SLC16A6	9120
226197_at	AR	367
232105_at	BLACAT1	1.02E+08
210347_s_at	BCL11A	53335
214053_at	ERBB4	2066
227498_at	SOX6	55553
204667_at	FOXA1	3169
213260_at	FOXC1	2296
228969_at	AGR2	10551

**Table 4:** Percentages of different model performances in both test and validation.

Model	Accuracy		Precision		Recall		F1 Score		AUC Score	
				
	Test	Validn	Test	Validn	Test	Validn	Test	Validn	Test	Validn
CTBC	93.74	93.56	98.05	99.2	89.32	87.9	93.48	93.21	97.11	96.46
RFC	93.02	91.59	97.64	94.66	88.26	88.26	92.71	91.34	96.56	96.29
MLP	91.59	92.67	92.09	96.15	91.1	88.97	91.59	92.42	97.05	96.16
XGBC	94.1	90.7	98.06	91.04	90.04	90.39	93.88	90.71	96.83	96.14
HGBC	93.2	91.95	98.41	96.83	87.9	86.83	92.86	91.56	96.82	95.67
SVC	90.34	89.62	94.86	93.39	85.41	85.41	89.89	89.22	95.8	94.35
GBC	91.95	87.84	93.7	87.9	90.04	87.9	91.83	87.9	96	93.38
ADBC	89.98	86.23	91.21	87.23	88.61	85.05	89.89	86.13	94.19	92.8

**Table 5:** The Cytohubba scores of the 7 hub genes.

Shared Name	Name	Degree	MCC	Closeness
9606.ENSP00000405330	ESR1	7.112	0.37904	2.64533
9606.ENSP00000250448	FOXA1	6.609	0.47564	2.38933
9606.ENSP00000368632	GATA3	6.294	0.47549	2.304
9606.ENSP00000216037	XBP1	6.151	0.51861	2.304
9606.ENSP00000370896	GREB1	6.033	0.58344	2.26133
9606.ENSP00000363822	AR	6.006	0.45378	2.26133
9606.ENSP00000391490	AGR2	5.732	0.32413	2.34667

## Data Availability

All the primary data used in the study were downloaded from the GDC data portal under the project named TCGA-BRCA (https://portal.gdc.cancer.gov/projects/TCGA-BRCA), and the NCBIGEO repository (https://www.ncbi.nlm.nih.gov/geo/) with accession numbers GSE7904, GSE21653, GSE43358, GSE45827, GSE65194, GSE76275, GSE83937, GSE95700, GSE103091, GSE135565, GSE157284, GSE167213, and GSE61724. **GSE7904**: RNA Microarray expression profiling of 62 human breast tissue samples including 43 tumors, 7 normal breast and 12 normal organelles. https://www.ncbi.nlm.nih.gov/geo/query/acc.cgi?acc=GSE7904 **GSE21653**: RNA Microarray expression profiling of 266 breast tissue samples of TNBC, non-TNBC, and normal cases. https://www.ncbi.nlm.nih.gov/geo/query/acc.cgi?acc=GSE21653 **GSE43358**: RNA Microarray expression profiling of 57 breast tumors representing TNBC and non-TNBC subtypes. https://www.ncbi.nlm.nih.gov/geo/query/acc.cgi?acc=GSE43358 **GSE45827**: RNA Microarray expression profiling of 155 samples representing 41 TNBC, 59 non-TNBC, 11 normal tissue samples and 14 cell lines. https://www.ncbi.nlm.nih.gov/geo/query/acc.cgi?acc=GSE45827 **GSE65194**: RNA Microarray expression profiling of 130 breast cancer samples (41 TNBC and 89 non-TNBC), 11 normal breast tissue samples and 14 TNBC cell lines. https://www.ncbi.nlm.nih.gov/geo/query/acc.cgi?acc=GSE65194 **GSE76275**: RNA Microarray expression profiling of 265 breast tissue samples including 198 TNBC and 67 non-TNBC tumors. https://www.ncbi.nlm.nih.gov/geo/query/acc.cgi?acc=GSE76275 **GSE83937**: RNA Microarray expression profiling of 131 TNBC samples. https://www.ncbi.nlm.nih.gov/geo/download/?acc=GSE83937&format=file **GSE95700**: RNA Microarray expression profiling of 57 TNBC samples. https://www.ncbi.nlm.nih.gov/geo/query/acc.cgi?acc=GSE95700 **GSE103091**: RNA Microarray expression profiling of 238 TNBC samples. https://www.ncbi.nlm.nih.gov/geo/query/acc.cgi?acc=GSE103091 **GSE135565**: RNA Microarray expression profiling of 84 TNBC samples https://www.ncbi.nlm.nih.gov/geo/query/acc.cgi?acc=GSE135565 **GSE157284**: RNA Microarray expression profiling of 82 TNBC samples. https://www.ncbi.nlm.nih.gov/geo/query/acc.cgi?acc=GSE157284 **GSE167213**: RNA Microarray expression profiling of 124 TNBC samples. https://www.ncbi.nlm.nih.gov/geo/query/acc.cgi?acc=GSE167213 **GSE61724:** The model validation dataset. https://www.ncbi.nlm.nih.gov/geo/query/acc.cgi?acc=GSE61724

## Abbreviations

TNBC: Triple-negative breast cancer
GEO: Gene Expression Omnibus
DEGs: Differentially Expressed Genes
LIMMA: Linear Model for Microarray Analysis
AI: Artificial Intelligence
ML: Machine Learning
BL1: Basal-like 1
BL2: Basal-like 2
M: Mesenchymal
MSL: Mesenchymal stem-like
LAR: Luminal Androgen Receptor
AR: Androgen Receptor
PM: Precision Medicine
PO: Precision Oncology
DFS: Disease-free survival
PARP: Poly ADP Ribose Polymerase Enzyme
sTILs: Stromal Tumor-infiltrating lymphocytes
RMA: Robust Multi-chip Average
logFC: Log Fold Change
RFC: Random Forest Classifier
RFE: Recursive Feature Elimination
RFECV: Recursive Feature Elimination with Cross-Validation
MIC: Mutual Information Classifier
MRMR: Minimal Redundancy Maximal Relevance
SVC: Support Vector Classifier
GBC: Gradient Boosting Classifier
HGBC: HistGradientBoosting Classifier
XGBC: eXtreme Gradient Boosting Classifier
CTBC: CatBoost Classifier
ADBC: AdaBoost Classifier
MLP: Multi-layer Perceptron
GO: Gene Ontology
FDR: False Discovery Rate
PPI: Protein–Protein Interaction
STRING: Search Tool for Retrieval of Interacting Genes
MCODE: Molecular Complex Detection
MF: Molecular Function
BP: Biological Process
CC: Cellular Component
MCC: Mean Clique Centrality
